# Efficacy, Safety, and Correlative Biomarkers of Toripalimab in Previously Treated Recurrent or Metastatic Nasopharyngeal Carcinoma: A Phase II Clinical Trial (POLARIS-02)

**DOI:** 10.1200/JCO.20.02712

**Published:** 2021-01-25

**Authors:** Feng-Hua Wang, Xiao-Li Wei, Jifeng Feng, Qi Li, Nong Xu, Xi-Chun Hu, Wangjun Liao, Yi Jiang, Xiao-Yan Lin, Qing-yuan Zhang, Xiang-Lin Yuan, Hai-Xin Huang, Ye Chen, Guang-Hai Dai, Jian-Hua Shi, Lin Shen, Shu-Jun Yang, Yong-Qian Shu, Yun-Peng Liu, Weifeng Wang, Hai Wu, Hui Feng, Sheng Yao, Rui-Hua Xu

**Affiliations:** ^1^ Department of Medical Oncology of Sun Yat-Sen University Cancer Center, State Key Laboratory of Oncology in South China, Collaborative Innovation Center of Cancer Medicine, Sun Yat-sen University, Guangzhou, China; ^2^ Jiangsu Cancer Hospital, Jiangsu Institute of Cancer Research, Nanjing, China; ^3^ Shanghai General Hospital, Shanghai Jiao Tong University School of Medicine, Shanghai, China; ^4^ The First Affiliated Hospital, School of Medicine, Zhejiang University, Hangzhou, China; ^5^ Fudan University Shanghai Cancer Center, Shanghai, China; ^6^ Cancer Center of Nan Fang Hospital, Guangzhou, China; ^7^ The Affiliated Cancer Hospital of Shantou University, Shantou, China; ^8^ Union Hospital of Fujian Medical University, Fuzhou, China; ^9^ The affiliated Cancer Hospital of Harbin Medical University, Harbin, China; ^10^ Wuhan Tongji Hospital, Wuhan, China; ^11^ Liuzhou Worker’s Hospital, Liuzhou, China; ^12^ Cancer Center of West China Hospital of Sichuan University, Chengdu, China; ^13^ Beijing 301 Hospital, Beijing, China; ^14^ Linyi Cancer Hospital, Linyi, China; ^15^ Beijing Cancer Hospital & Institute, Beijing, China; ^16^ Henan Cancer Hospital, Zhengzhou, China; ^17^ Jiangsu Provincial Hospital, Nanjing, China; ^18^ The First Hospital of China Medical University, Shenyang, China; ^19^ OrigiMed, Shanghai, China; ^20^ Shanghai Junshi Biosciences Co, Ltd, Shanghai, China; ^21^ Research Unit of Precision Diagnosis and Treatment for Gastrointestinal Cancer, Chinese Academy of Medical Sciences, Guangzhou, China

## Abstract

As yet, no checkpoint inhibitor has been approved to treat nasopharyngeal carcinoma (NPC). This study was aimed to evaluate the antitumor activity, safety, and biomarkers of toripalimab, a new programmed death-1 (PD-1) inhibitor for recurrent or metastatic NPC (RM-NPC) refractory to standard chemotherapy.

**PATIENTS AND METHODS:**

In this single-arm, multicenter phase II study, patients with RM-NPC received 3 mg/kg toripalimab once every 2 weeks via intravenous infusion until confirmed disease progression or unacceptable toxicity. The primary end point was objective response rate (ORR). The secondary end points included safety, duration of response (DOR), progression-free survival (PFS), and overall survival (OS).

**RESULTS:**

Among all 190 patients, the ORR was 20.5% with median DOR 12.8 months, median PFS 1.9 months, and median OS 17.4 months. Among 92 patients who failed at least two lines of systemic chemotherapy, the ORR was 23.9%. The ORRs were 27.1% and 19.4% in PD-L1+ and PD-L1− patients, respectively (*P* = .31). Patients with ≥ 50% decrease of plasma Epstein-Barr virus (EBV) DNA copy number on day 28 had significantly better ORR than those with < 50% decrease, 48.3% versus 5.7% (*P* = .0001). Tumor mutational burden had a median value of 0.95 muts/mega-base in the cohort and had no predictive value for response. Whole-exome sequencing results from 174 patients revealed that the patients with genomic amplification in *11q13* region or *ETV6* genomic alterations had poor responses to toripalimab.

**CONCLUSION:**

The POLARIS-02 study demonstrated a manageable safety profile and durable clinical response of toripalimab in patients with chemorefractory metastatic NPC. An early decrease in plasma EBV DNA copy number correlated with favorable response.

## INTRODUCTION

Nasopharyngeal carcinoma (NPC) is distinctive from other head and neck cancers with unique geographical, etiological, and biological features.^1^ It is distributed predominantly in South China, Southeast Asia, Middle East, and North Africa.^1-3^ Nonkeratinizing NPC is the most common subtype in both North America and Southeast Asia,^4,5^ which is more closely associated with Epstein-Barr virus (EBV) infection, more responsive to radiation therapy, and has better survival than keratinizing NPC.^5^

**CONTEXT**

**Key Objective**
There are no approved therapies for the later-line treatment of nasopharyngeal carcinoma (NPC), and no immune checkpoint inhibitor has been approved for the treatment of NPC.
**Knowledge Generated**
Toripalimab showed a manageable safety profile and a response rate of 20.5% with durable responses. Changes in plasma Epstein-Barr virus DNA copy number correlated with response. Genomic alternations in *11q13* region or *ETV6* gene may be associated with low response rates.
**Relevance**
This study supports the use of toripalimab as a treatment option for recurrent or metastatic NPC in heavily pretreated patients.

Patients with recurrent or metastatic NPC (RM-NPC) have poor prognosis, with median overall survival (OS) <20 months.^6^ Platinum doublet, like cisplatin plus gemcitabine, is the standard first-line treatment for RM-NPC.^7^ However, there is no standard treatment option in second-line settings and beyond.^6^

EBV infection is instrumental in NPC development.^8^ EBV-induced NPCs often present with intensive lymphocyte infiltration and overexpression of programmed death ligand-1 (PD-L1),^9^ indicating the potential application of programmed death-1 (PD-1) blockade immunotherapy. Several small size phase II studies of anti-PD-1 immune checkpoint inhibitors (ICIs) in patients with RM-NPC showed objective response rate (ORR) from 20.5% to 34.1%.^10-12^ However, predictive biomarkers for ICI therapy in NPC had not been established.^10-12^

Toripalimab, a humanized IgG_4_ monoclonal antibody against PD-1,^13^ was first approved in December 2018 for the second-line treatment of advanced melanoma in China.^14^ Here, we report the result of phase II pivotal study (POLARIS-02) evaluating the efficacy and safety of toripalimab in previously treated RM-NPC. It was to date the largest prospective study of anti-PD-1 monotherapy in chemorefractory NPC. Potential efficacy predictors were explored to identify the NPC population most likely to respond to ICI therapy.

## PATIENTS AND METHODS

### Patients and Study Design

This study is the NPC cohort from a phase Ib/II, multicohort trial (ClinicalTrials.gov identifier: NCT02915432) evaluating the safety and clinical activity of toripalimab in patients with RM-NPC, head and neck cancer, gastric cancer, and esophageal cancer. The study Protocol was approved by institutional ethics committees of all participating centers. This study was conducted in accordance with the Declaration of Helsinki and the international standards of good clinical practice.

Key eligibility criteria included histologically or cytologically documented RM-NPC refractory to prior standard chemotherapy, or disease progression within 6 months after adjuvant chemotherapy or chemoradiotherapy, age 18 years or older, measurable disease, Eastern Cooperative Oncology Group performance status of 0 or 1, and adequate organ function. Key exclusion criteria included anticancer monoclonal antibody therapy within 4 weeks before treatment initiation, any anticancer therapy within 2 weeks before treatment initiation, prior ICI treatment, systemic corticosteroid therapy within 7 days before treatment initiation, known additional malignancies, and active CNS metastases.

The definition of patient population with at least two prior lines (2L+) of systemic chemotherapy included: (1) received at least two lines of systemic chemotherapy; (2) first-line chemotherapy must include platinum-based regimen; (3) neoadjuvant, adjuvant, or concurrent chemoradiotherapy were considered as a line of systemic treatment if tumor recurrence or metastasis occurred within 6 months after the end of neoadjuvant, adjuvant, or concurrent chemotherapy; (4) stage IVb at enrollment as defined by Union for International Cancer Control and American Joint Committee on Cancer staging system for NPC, seventh edition; (5) clear evidence of disease progression to prior therapy at enrollment. Intolerance to chemotherapy should not be counted as a line of systemic treatment.

### Treatment and Assessments

Patients received toripalimab 3 mg/kg once every 2 weeks via intravenous infusion until disease progression, intolerable toxicity, or voluntary withdrawal of informed consent. Patients experiencing initial disease progression could continue to receive toripalimab under the condition of potential benefit to the patients, with the consensus of investigator and sponsor.

Tumor response was assessed according to RECIST v1.1 and immune-related RECIST by independent radiologic review committee (IRC) and investigators. Adverse events were graded according to National Cancer Institute Common Terminology Criteria version 4.0.

### Study End Points

The primary end point was ORR determined by IRC according to RECIST v1.1. The secondary end points included safety, duration of response (DOR), disease control rate (DCR), progression-free survival (PFS), and OS. Exploratory end points included PD-L1 expression, plasma EBV DNA copy number, tumor mutational burden (TMB), and genetic biomarkers by whole-exome sequencing (WES) as potential efficacy predictors.

PD-L1 expression was determined by immunohistochemistry (IHC) staining with SP142 antibody^15^ in a central lab and evaluated by certified pathologists. PD-L1 expression on tumor cells (TCs, defined as tumor proportion score) and immune cells (ICs) were evaluated.

Plasma EBV DNA copy number was determined by the quantitative reverse transcription polymerase chain reaction (qRT-PCR) method with probes against EBV genes before treatment and every 4 weeks until disease progression.

WES was performed using SureSelect Human All Exon V6 kit (Agilent, Santa Clara, CA) on tumor and matched blood samples. Genomic alterations including microsatellite stability status, nucleotide variants, short and long insertions and deletions (INDELs), copy number variants, and gene rearrangement and fusions were assessed. TMB was determined by analyzing somatic mutations including coding base substitutions and INDELs according to mega-base (Mb).

### Sample Size Determination and Statistical Analysis

The sample size of the original NPC cohort was estimated according to Simon's two-stage design. In stage I, among 34 evaluable patients with NPC, if the number of responders was <2, the study cohort would be terminated. Otherwise, an additional 14 patients would be enrolled. Considering a dropout rate of 10%, a total of 54 patients with NPC would be enrolled. The study Protocol was amended to increase the NPC cohort size to 100 after a promising result (26.5% ORR) among the first 34 patients at stage I. The sample size of the NPC cohort was further amended to 180 patients after a type-B meeting with the National Medical Product Administration (NMPA) of China for registration purpose and was based on the following assumptions. At a one-sided significance level of 0.025, 160 patients could provide at least 80% power to show the efficacy of toripalimab at targeted ORR of 24% versus 15% for alternative third-line therapy using Clopper-Pearson method. As an estimated 20 patients with one prior line of systemic treatment had been enrolled in the cohort, a total of 180 patients would be enrolled.

Safety analysis included all patients with at least one dose of study drug. ORR and its 95% exact CI were determined by Clopper and Pearson methodology. Fisher's exact test was used to compute two-tailed *P* values from contingency tables. PFS and OS were plotted using the Kaplan-Meier method, with median and corresponding two-sided 95% CI. Statistics analyses were performed using SAS version 9.4 or GraphPad Prism software.

## RESULTS

### Patient Population

**FIG A1.**
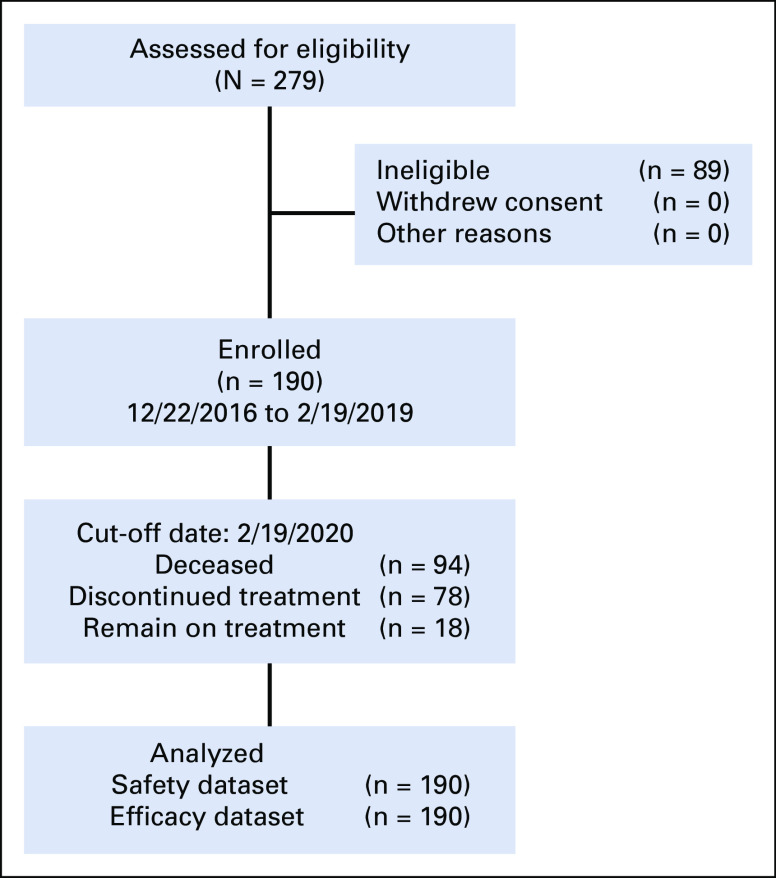
Consort diagram for the phase II study of toripalimab treating patients with recurrent or metastatic nasopharyngeal carcinoma refractory to standard therapy.

From December 2016 to February 2019, 17 participating centers in China screened 279 patients with RM-NPC and 190 patients were enrolled (Appendix Fig A1, online only). Ninety-two patients (48.4%) had at least two prior lines of systemic chemotherapy. Baseline characteristics are summarized in Table 1. According to histology subtypes (WHO Classification of Tumors), 182 patients (95.8%) were nonkeratinizing and 8 patients (4.2%) were keratinizing NPC.

**TABLE 1.**
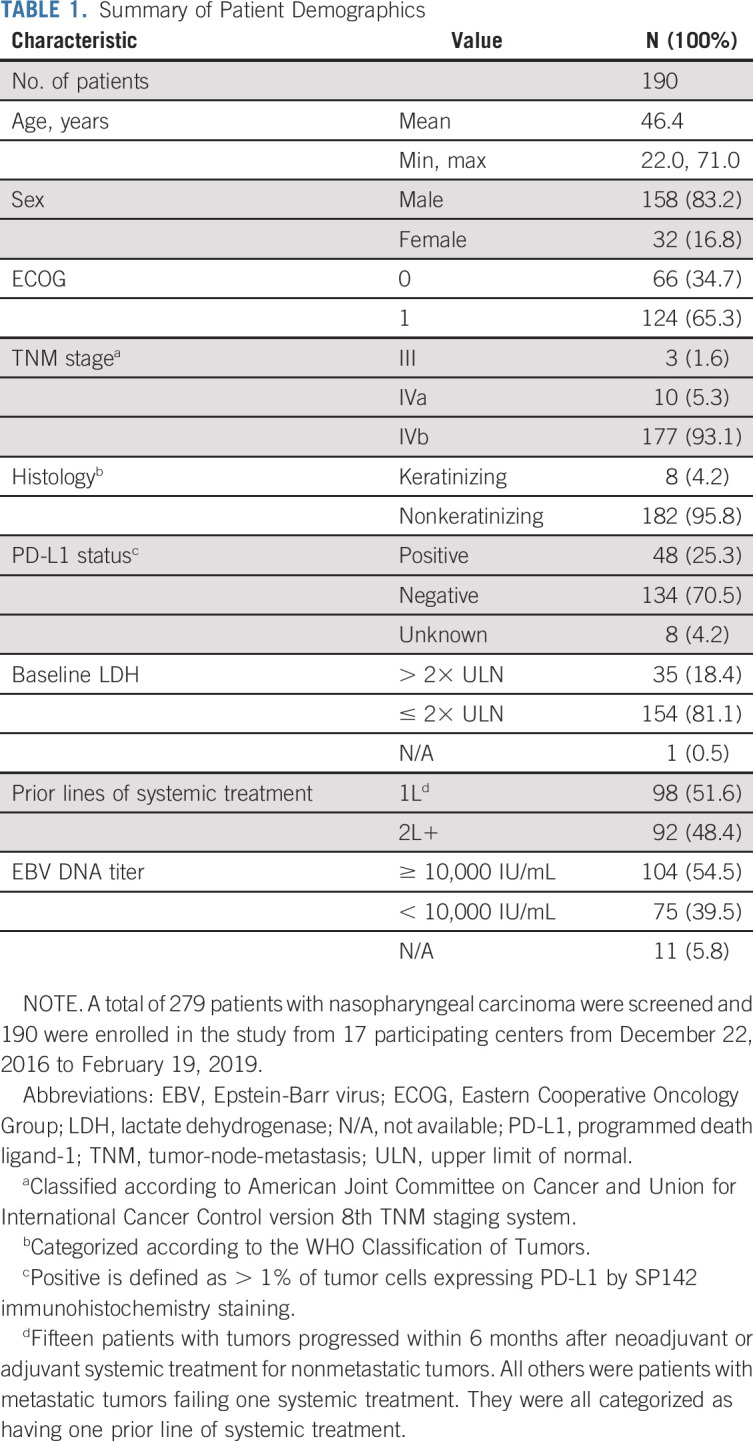
Summary of Patient Demographics

### Safety

All enrolled patients received a median of eight doses of toripalimab (range, 1-69 doses). Permanent discontinuation of toripalimab as a result of treatment-related adverse event (TRAE) occurred in four patients, and dose interruption as a result of TRAE occurred in seven patients.

TRAEs occurred in 141 (74.2%) patients. Common TRAEs (> 5%) were listed in Table 2. Grade 3-5 TRAEs occurred in 27 (14.2%) patients (Appendix Table A1, online only). Main immune-related adverse events included hypothyroidism (n = 45, 23.7%), hyperthyroidism (n = 5, 2.6%), abnormal liver function (n = 3, 1.6%), interstitial lung disease (n = 3, 1.6%), dermatomyositis (n = 1, 0.5%), and autoimmune myocarditis (n = 1, 0.5%).

**TABLE 2.**
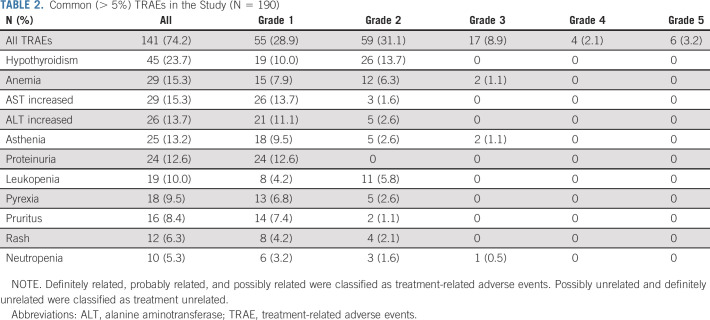
Common (> 5%) TRAEs in the Study (N = 190)

### Antitumor Activity

**FIG 1.**
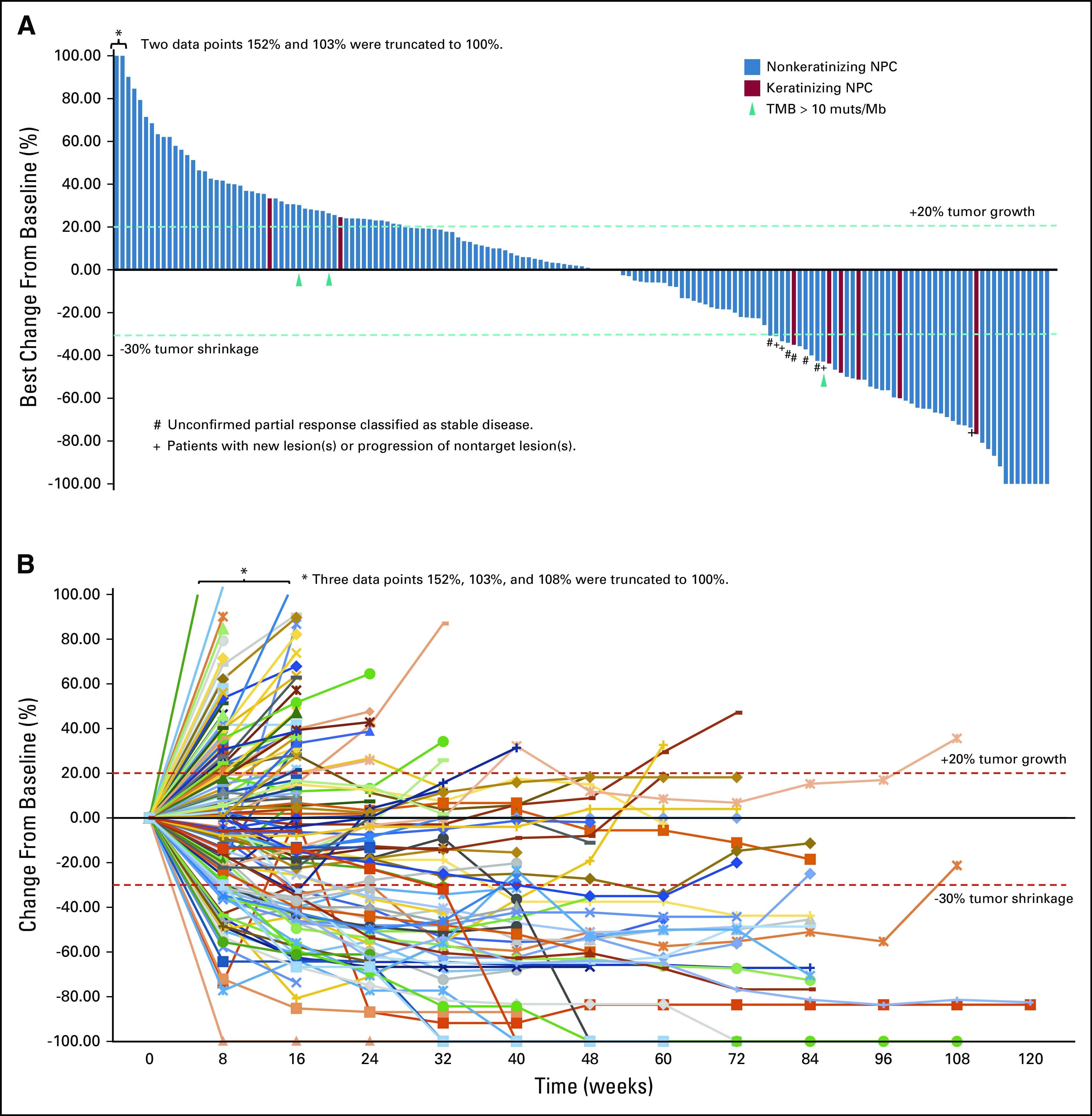
Tumor responses of patients with nasopharyngeal carcinoma in this study cohort. (A) Maximal change of tumor size from baseline assessed by independent review committee (IRC) according to RECIST v1.1 for patients with at least one post-treatment radiographic evaluation. The length of the bar represents maximal decrease or minimal increase in target lesion(s). (B) Change of individual tumor burden over time from baseline assessed by IRC according to RECIST v1.1. Tumor response was assessed before treatment, once every 8 weeks in the first year and then once every 12 weeks from the second year until disease progression.

As of February 19, 2020, 1 year after the last enrollment, 94 (49.5%) patients died, 78 (41.1%) stopped treatment, and 18 (9.5%) remained on treatment. Median treatment duration was 3.7 months (range, 0.2-34.8 months). Among 190 patients assessed by IRC according to RECIST v1.1, the ORR was 20.5% (95% CI, 15.0 to 27.0), and the DCR was 40.0% (95% CI, 33.0 to 47.3) (Table 3). Any decrease in target lesions from baseline was observed in 73 (38.4%) patients (Fig 1). The median time to response was 1.8 months (95% CI, 1.8 to 2.1) and the median DOR was 12.8 (95% CI, 9.4 to not estimable) months. The median PFS was 1.9 (95% CI, 1.8 to 3.5) months and the median OS was 17.4 (95% CI, 11.7 to 22.9) months. The median OS of patients with objective response (n = 39) or stable disease (n = 38) was not reached by the cutoff date, whereas the median OS of patients with progressive disease (n = 113) was 8.4 months.

**TABLE 3.**
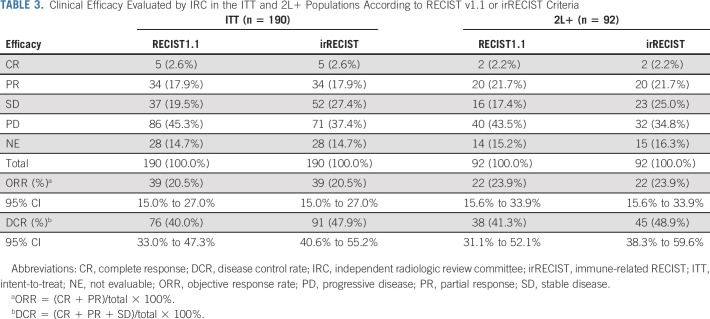
Clinical Efficacy Evaluated by IRC in the ITT and 2L+ Populations According to RECIST v1.1 or irRECIST Criteria

For 92 2L+ patients, the ORR was 23.9% (95% CI, 15.6 to 33.9), and the DCR was 41.3% (95% CI, 31.1 to 52.1) as assessed by IRC according to RECIST v1.1. The median DOR was 21.5 (95% CI, 7.7 to not estimable) months, the median PFS was 2.0 (95% CI, 1.8 to 3.6) months and the median OS was 15.1 (95% CI, 10.4 to 20.4) months.

Subgroup analysis of both intent-to-treat population and 2L+ population showed that female patients, patients with age ≥ 60 years, lactate dehydrogenase ≤ 2× upper limit of normal, or without liver metastasis had numerically higher ORR (Appendix Tables A2 and A3, online only). None of the differences, however, were statistically significant.

Interestingly, compared with nonkeratinizing NPC (n = 182), keratinizing NPC (n = 8) had an ORR of 62.5%. However, given the small sample size, further study is warranted in a larger cohort of keratinizing NPC.

### Plasma EBV DNA

Patients with EBV DNA titer <10,000 IU/mL (n = 75, 54.5%) had numerically higher ORR than those with ≥ 10,000 IU/mL (n = 104, 39.5%), 26.7% versus 15.4%. But the difference was not statistically significant (*P* = .088) (Appendix Table A2).

**FIG 2.**
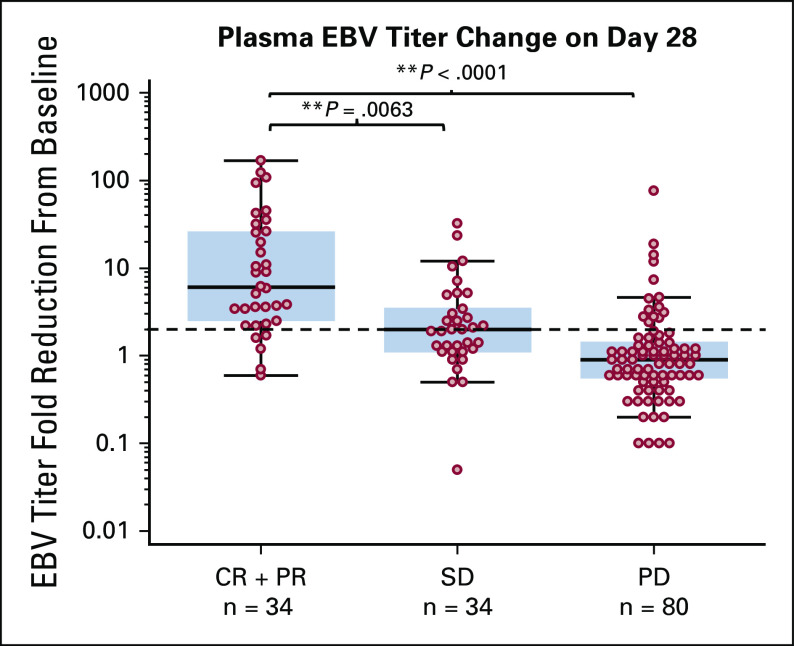
Plasma EBV DNA copy number was measured from 148 patients at baseline and on day 28. The fold reduction of EBV titer from baseline to day 28 was shown in patients with objective response (n = 34), stable disease (n = 34), or progressive disease (n = 80). T-test was used to determine statistical significance between two groups. Significant differences were observed between CR + PR and stable disease groups and CR + PR and progressive disease groups. Fold reduction = (baseline EBV tier)/(day 28 EBV titer). CR, complete response; EBV, Epstein-Barr virus; PR, partial response.

Dynamic monitoring of plasma EBV DNA copy number was performed during the study, and results were available from 148 patients. Patients with objective responses had a significant greater EBV titer decrease from baseline to day 28 than patients with stable disease or progressive disease (Fig 2). Furthermore, patients with ≥ 50% EBV titer decrease on day 28 (n = 60) had significantly higher ORR than those with <50% decrease (n = 88) (48.3% *v* 5.7%, *P* = .0001). Notably, 14 patients who responded to toripalimab and later experienced disease progression had at least 100% increase of plasma EBV titer occurring at a median of 3 months before radiographic identification of disease progression.

### PD-L1 Expression

**FIG A2.**
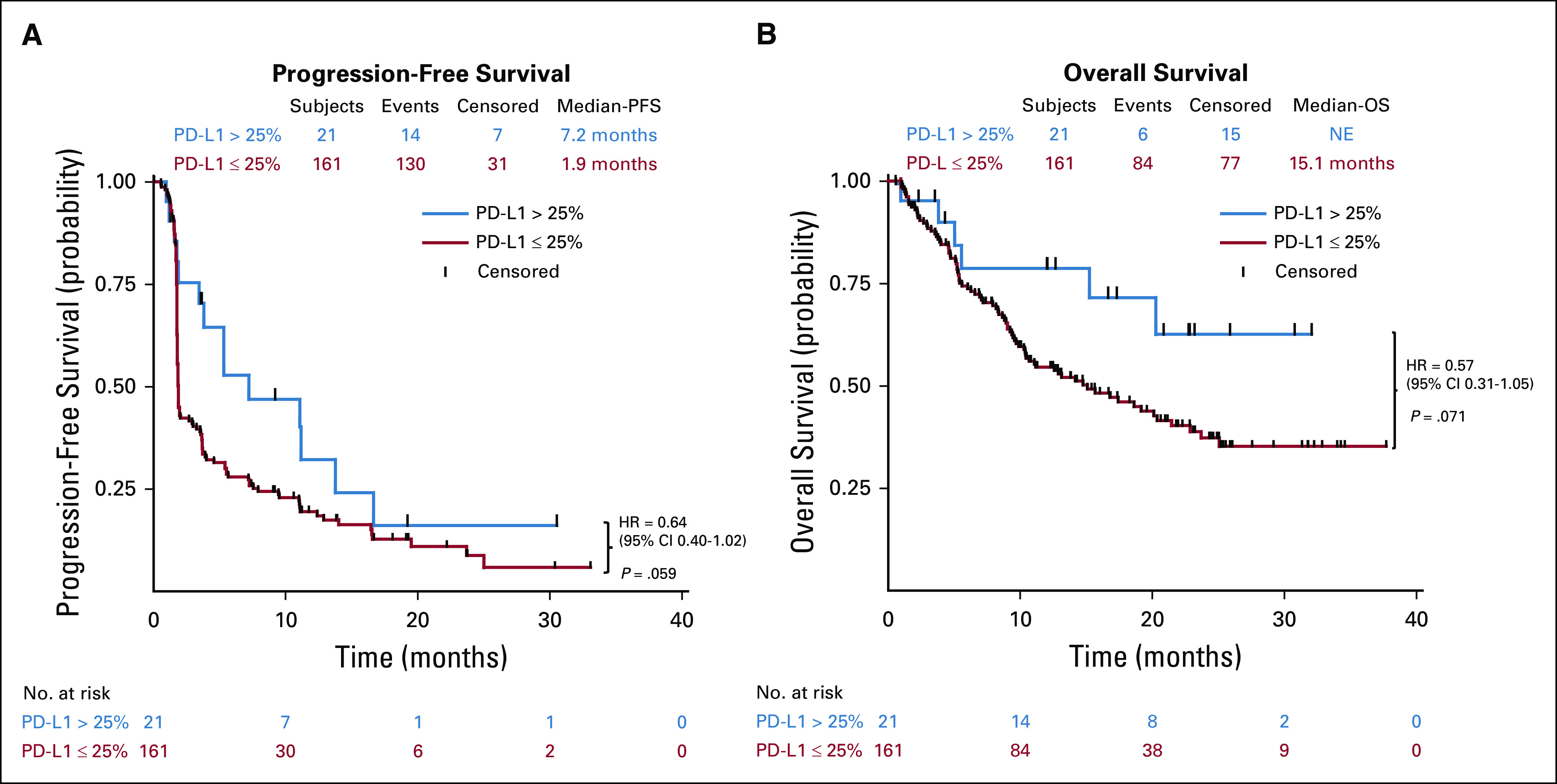
Patient survival in relation to tumor PD-L1 expression. (A) Progression-free survival of PD-L1 > 25% versus PD-L1 ≤ 25% patients. (B) Overall survival of PD-L1 > 25% versus PD-L1 ≤ 25% patients. Percentages of survival patients are shown at indicated time points. Censored patients are marked with “┃” in the graph. Numbers of patients at risk at indicated time points are shown below the x-axis. PFS was defined as the time from first dosing to first recorded progression of disease or death because of any reason, whichever came first. Patients with no PFS event during follow-up will be censored at the last tumor evaluation. Patients with no postbaseline evaluation will be censored at the date of first dosing. Patients with death or progression after two or more consecutive missing tumor assessments were censored at the last postbaseline tumor evaluation before the missed visits. If patients did not have the postbaseline tumor evaluation before the missed visits, they were censored at the date of first dosing. OS was defined as the time from first dosing to death because of any cause. Patients with no OS events were censored on the last known survival date. PD-L1, programmed death ligand-1; PFS, progression-free survival.

Among 190 patients, 48 (25.3%) were PD-L1+ and 134 (70.5%) were PD-L1− defined by SP142 IHC staining, whereas eight (4.2%) patients had unknown PD-L1 status. PD-L1+ patients, defined by TC positive staining > 1%, had numerically higher ORR than PD-L1− patients (27.1% *v* 19.4%), but the difference was not statistically significant (*P* = .31). A total of 21/190 (11.1%) patients were identified as PD-L1 high expression (> 25%). Patients with PD-L1 high expression had a higher ORR (38.1% *v* 19.3%), better median PFS (7.2 months *v* 1.9 months) and median OS (unreached *v* 15.1 months) than patients with PD-L1 low expression. However, none of the differences were statistically significant (Appendix Fig A2, online only). PD-L1 expression defined by IC positive staining > 1% was also evaluated. PD-L1 IC+ patients (n = 39) had 23.1% ORR, whereas PD-L1 IC− patients (n = 143) had 21.0% ORR (Appendix Table A4, online only).

### Genomic Mutational Analysis

**FIG A3.**
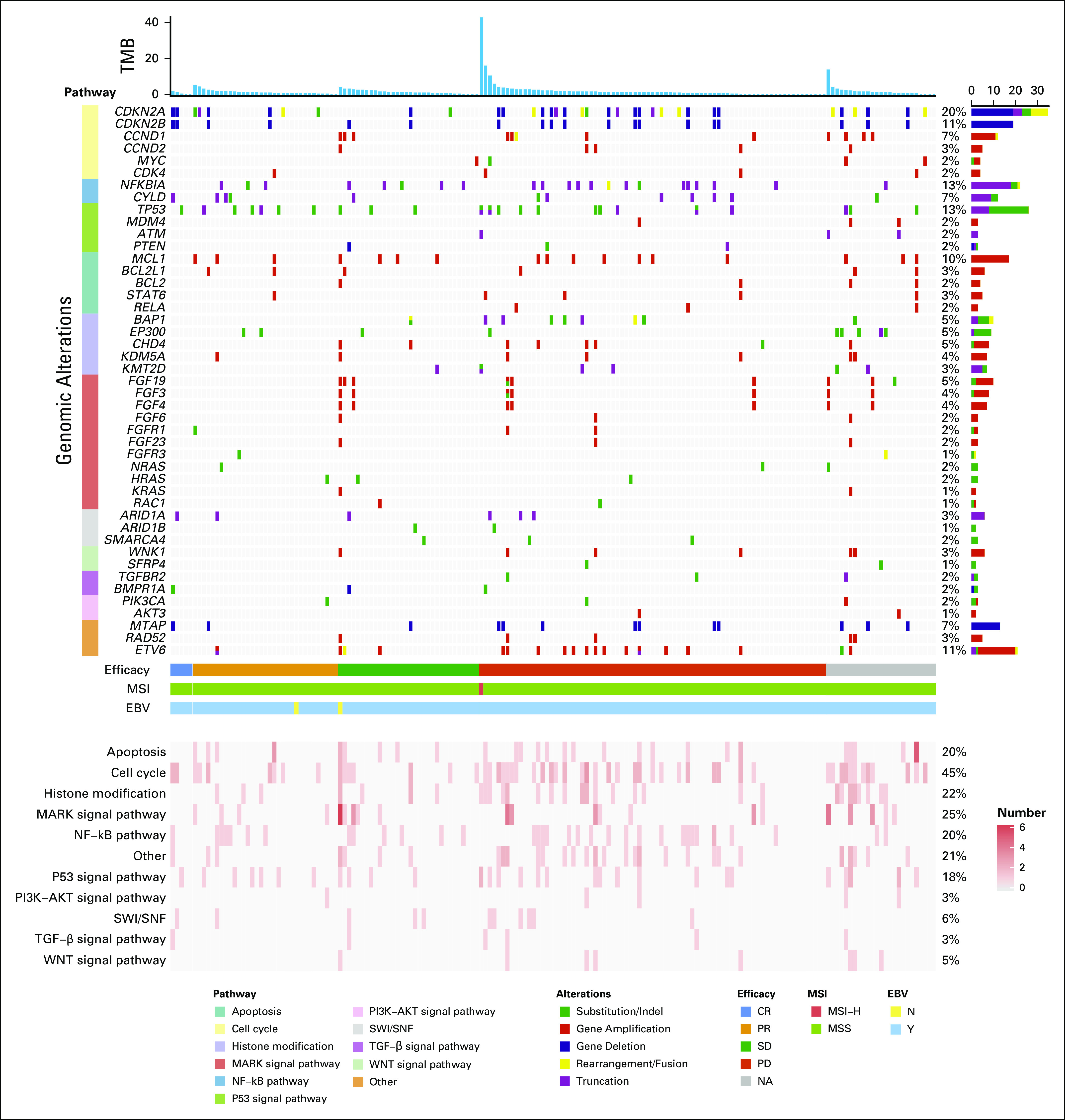
Genetic alternations, frequencies, and pathway alternations identified by whole-exome sequencing from 174 available patients with nasopharyngeal carcinoma. Patients were grouped by clinical responses. CR, complete response; EBV, Epstein-Barr virus; MSI, microsatellite instability; MSS, microsatellite stable; PR, partial response; PD, progressive disease; SD, stable disease; SWI/SNF: SWItch/Sucrose Non-Fermentable complexes.

WES results were available for 174 patients. The most frequently altered genes (≥ 10%) identified in this study included *CDKN2A* (20%), *TP53* (13%), *NFKB1A* (13%), *CDKN2B* (11%), *ETV6* (11%), and *MCL1* (10%) (Appendix Fig A3, online only).

None of the genomic alternations had statistically significant association with clinical efficacy (partial response [PR] + complete response [CR] *v* stable disease + progressive disease). Notably, 12 patients with genomic amplification in the *11q13* region (including *CCND1*, *FGF14*, *FGF3*, and *FGF4* genes) had an ORR of 0%, whereas 19 patients with *ETV6* genomic alternations (including 17 amplifications) had an ORR of 5.3%.

### TMB

**FIG A4.**
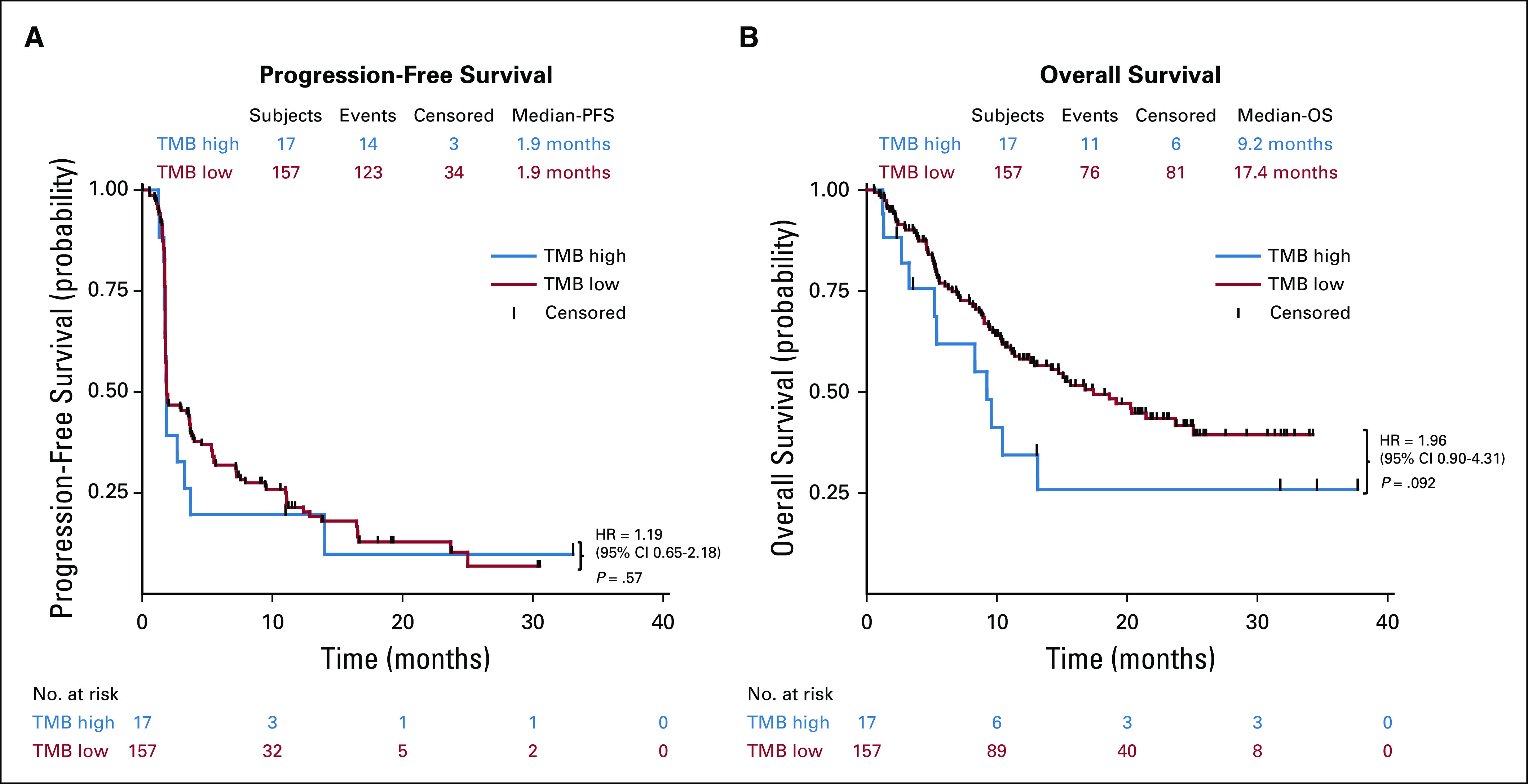
Patient survival in relation to tumor mutational burden. The tumor mutational burden (TMB) was calculated by summing up somatic mutations within the coding regions by whole-exome sequencing. Using top 10% TMB value of 2.9 mutations per Mb as a cutoff. (A) Progression-free survival of TMB ≥ 2.9 muts/Mb versus TMB < 2.9 muts/Mb patients. (B) Overall survival of TMB ≥ 2.9 muts/Mb versus TMB < 2.9 muts/Mb patients. Percentages of survival patients are shown at indicated time points. Censored patients are marked with “┃” in the graph. Numbers of patients at risk at indicated time points are shown below the x-axis.

TMB determined by WES had a median value of 0.95 muts/Mb in the cohort. As suggested by Robert M. Samstein et al,^16^ a cutoff of the top 10% of TMB value (2.9 muts/Mb) was selected. Patients with TMB ≥ 2.9 muts/Mb and those with TMB < 2.9 muts/Mb had no significant differences in ORR, PFS, and OS (Appendix Fig A4, online only). Four patients had TMB value over 10 muts/Mb in this cohort, including one microsatellite instability-high (MSI-H) patient. Surprisingly, none of them had an objective response to toripalimab with short PFS (1.68-3.25 months) and OS (2.30-9.56 months).

## DISCUSSION

NPC is distinct in its anatomic location and biology from other epithelial tumors in the head and neck region. No ICI has been approved to treat NPC. Phase III clinical trials which led to the approval of pembrolizumab or nivolumab for head and neck squamous cell carcinoma (HNSCC) specifically excluded NPC.^17,18^ Standard treatment for patients with RM-NPC beyond the first-line setting has not been established. Chemotherapeutic or targeted agents for 2L+ patients only had moderate antitumor activity with ORRs ranging from 0% to 48% with median PFS of 5.2-5.4 months and median OS of 11.5-12.5 months.^6^ Several studies had shown promising activities of anti-PD-1 monotherapy for chemorefractory NPCs. Among 44 unselected advanced patients with NPC treated with nivolumab, the ORR was 20.5%.^10^ In KEYNOTE-028 study, 19 patients with PD-L1+ NPC treated with pembrolizumab had an ORR of 26.3%.^11^ Among 91 unselected advanced patients with NPC treated with camrelizumab, the ORR was 34.1%.^12^

In this study, with the largest NPC cohort treated with anti-PD-1 monotherapy to date, the ORR of toripalimab for all 190 patients was 20.5% (95% CI, 15.0 to 27.0), the response was durable with median DOR of 12.8 months and the median OS was 17.4 months. The definition of patient population with at least two prior lines of systemic chemotherapy (2L+) was clarified and redefined retrospectively by the Chinese regulatory agency, the NMPA, and 92 patients qualified as 2L+. Among 2L+ patients, toripalimab had an ORR of 23.9% (95% CI, 15.6 to 33.9), with a median DOR of 21.5 months and a favorable median OS of 15.1 months.

There are three histopathological types of NPC by WHO classification, keratinizing squamous cell carcinoma, nonkeratinizing carcinoma, and basaloid squamous cell carcinoma. Nonkeratinizing subtype is more common in Southeast Asia and closely associated with EBV infection, whereas keratinizing subtype is associated with environmental factors, such as smoking and alcohol intake.^19^ The frequency of keratinizing subtype is about 2% in Southeast Asia, whereas it accounts for about 25% of all NPC in North America.^4,5^ Keratinizing NPC (n = 8) had a 62.5% ORR in this study. Although it is interesting, considering the small sample size and possible selection bias, further study in a larger cohort of keratinizing NPC is warranted.

Virus DNA has been recognized as a prognostic biomarker in several virus-mediated malignancies. Latent infection with EBV is crucial to the development of NPC.^20^ Measurement of plasma EBV DNA copy number has been recommended for NPC diagnostic and prognostic monitoring.^21-23^ The dynamic change of EBV titer is closely related to response to chemotherapy or radiotherapy.^24,25^ Similarly, plasma HPV DNA was indicated as a promising biomarker for monitoring chemoradiotherapy response in patients with HPV-associated oropharyngeal squamous cell carcinoma.^26^ However, the value of circulating viral DNA titer for immunotherapy has not been comprehensively investigated. In the current study, we found that patients with baseline EBV titer <10,000 IU/mL had numerically higher ORR than those with EBV titer ≥ 10,000 IU/mL (54.5% *v* 39.5%, *P* = .088). Notably, responding patients (CR or PR) had more significant EBV titer reduction from baseline to day 28 than patients with stable disease or progressive disease (Fig 2). Furthermore, patients with ≥ 50% decrease of EBV titer on day 28 had significantly better ORR than those with <50% decrease (ORR 48.3% *v* 5.7%, *P* = .0001). The results supported the positive association of plasma EBV DNA copy number reduction with improved disease control in response to immunotherapy.

PD-L1 expression status correlated with favorable response to immunotherapy in various cancers, but its value for NPC was unclear. Consistent with nivolumab in advanced NPC,^10^ our study observed a numerically higher but not statistically significant ORR of PD-L1 TC+ patients than PD-L1 TC− patients (27.1% *v* 19.4%). Although PD-L1 staining by SP142 on IC was used in other indications,^27^ we found that PD-L1 IC expression had no apparent association with clinical efficacy in our study, suggesting different PD-L1 expression patterns and applications to predict clinical response in different cancer types. The high heterogeneity of PD-L1 expression in NPC^28-30^ may also limit its value as a robust biomarker.

We also evaluated the predictive value of tumor TMB for RM-NPC in response to ICI therapy. Our study discovered that with a cutoff value of 2.9 muts/Mb (top 10% TMB value), TMB has no predictive value for clinical response. The median TMB value was 0.95 muts/Mb in the cohort, which was consistent with previous reports.^31,32^ It was suggested that rather than the accumulation of DNA mutations, EBV infection might play a more important role in driving NPC tumorigenesis.^33^ Consistent with our study, the results of KEYNOTE-012 study and KEYNOTE-055 study also found that TMB was less predictive for HNSCC patients with positive HPV or EBV, whereas high TMB correlated with a better response for HNSCC patients with negative HPV or EBV.^34,35^

WES was performed to identify genomic biomarkers for immunotherapy in patients with RM-NPC. The frequently altered genes were consistent with previous reports.^36,37^ Patients with genomic amplification in *11q13* region had no response and those with *ETV6* genomic alterations had an ORR of 5.3%, indicating potential resistance mechanisms to immunotherapy. Genomic amplification in *11q13* region was previously reported to associate with poor response to toripalimab in melanoma^14^ and esophageal squamous cell carcinoma.^38^ Overexpression of *CCND1*, *FGF*, and *ETV6* genes has been correlated with poor prognosis in patients with NPC.^39,40^ The impact of genomic amplification in *11q13* region and *ETV6* on immune function and immunotherapy needs to be further investigated.

In summary, POLARIS-02, the largest prospective clinical study of anti-PD-1 monotherapy in patients with chemorefractory RM-NPC, showed a manageable safety profile. The response rate and, more importantly, the DOR have demonstrated that toripalimab provides substantial benefit to patients with metastatic NPC who are receiving later-line therapy. This study also provided new perspectives on predictive biomarkers in NPC. Plasma EBV DNA copy number change might serve as a feasible predictor for clinical efficacy. A confirmatory phase III trial comparing toripalimab versus placebo in combination with cisplatin plus gemcitabine as first-line treatment for RM-NPC is ongoing (ClinicalTrials.gov identifier: NCT03430297).

## Figures and Tables

**TABLE A1.**
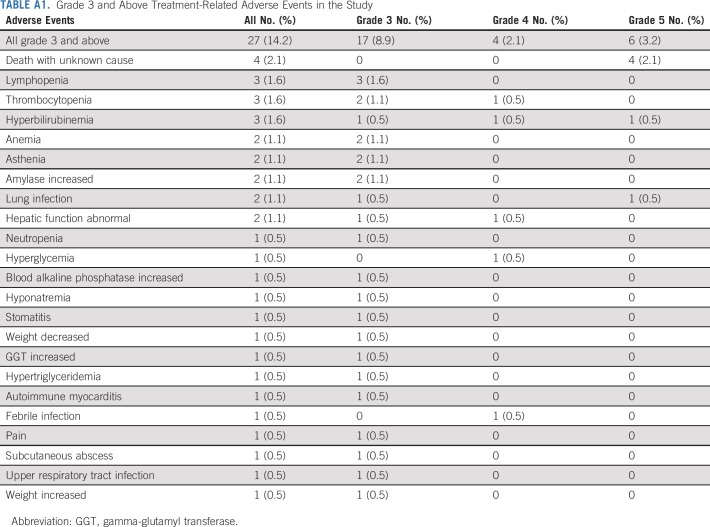
Grade 3 and Above Treatment-Related Adverse Events in the Study

**TABLE A2.**
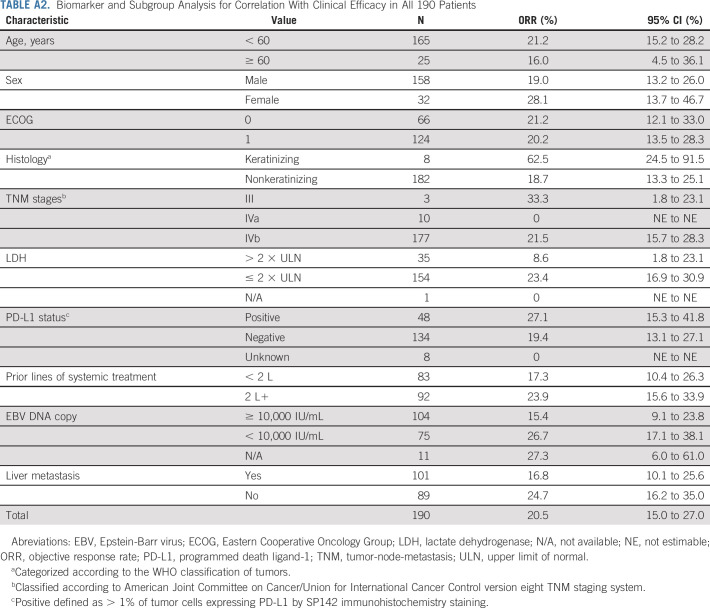
Biomarker and Subgroup Analysis for Correlation With Clinical Efficacy in All 190 Patients

**TABLE A3.**
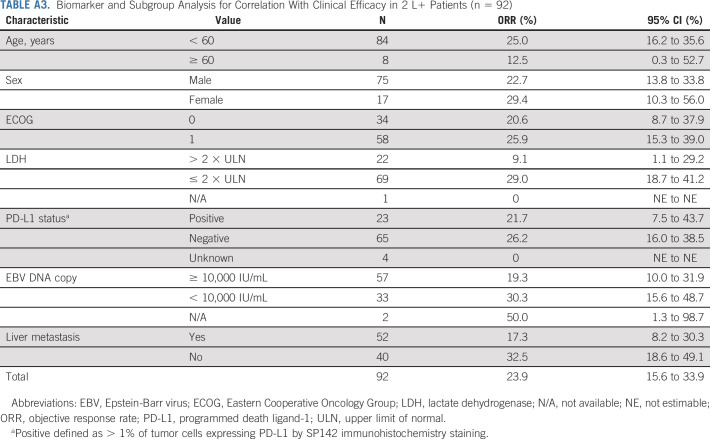
Biomarker and Subgroup Analysis for Correlation With Clinical Efficacy in 2 L+ Patients (n = 92)

**TABLE A4.**
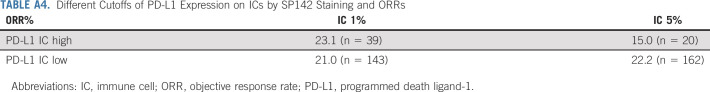
Different Cutoffs of PD-L1 Expression on ICs by SP142 Staining and ORRs
